# Intraductal papillary mucinous tumor of bile ducts radiologic and pathologic features: a case report

**DOI:** 10.1186/1757-1626-1-319

**Published:** 2008-11-17

**Authors:** Gianpaolo Carrafiello, Elena Bertolotti, Fausto Sessa, Tamara Cafaro, Gianlorenzo Dionigi, Eugenio Genovese, Renzo Dionigi, Carlo Fugazzola

**Affiliations:** 1 Department of Radiology, University Hospital of Insubria, Viale Borri 57, 21100 Varese, Italy; 2 Department of Pathology, University Hospital of Insubria, Via O. Rossi 9, 21100 Varese, Italy; 3 Department of Surgery, University Hospital of Insubria, Viale Borri 57, 21100 Varese, Italy

## Abstract

We report a case of a 67-year-old Caucasian man with right upper quadrant abdominal pain. He underwent radiologic investigations that revealed a solid, focal mass, at the V hepatic segment. Because a definitive diagnosis, based on imaging appearance of the lesion, was impossible in our case, we performed a hystopathological investigation but the biopsies were inconclusive. So, the definitive diagnosis of intraductal papillary mucinous tumor of bile ducts was made on surgical resected material.

Intraductal papillary neoplasm of the liver (IPNL) is a recently recognized entity which closely resembles an intraductal papillary mucinous tumor (IPMT) of the pancreas.

## Background

Papillary tumors of the intrahepatic bile ducts are characterized by intraluminal growth, sometimes in papillary masses with bile duct obstruction and dilatation. Papillary tumors generally produce a large amount of mucin and so they may occasionally impede the flow of the bile juice and cause a severe ductal dilatation. Intraductal papillary neoplasm of the liver (IPNL) is a recently recognized entity frequently reported in association with hepatolithiasis and resembling intraductal papillary mucinous tumor (IPMT) of the pancreas, in terms of its histopathology, pathophysiology, and excessive mucin production [1-5].

## Case report

**Figure 1:**
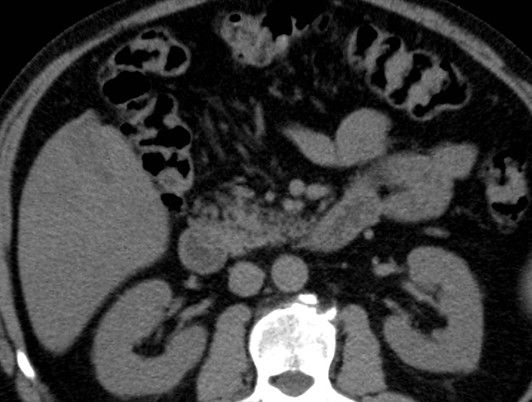
**Transverse CT scan**. It reveals a solid focal mass, about 3 cm in diameter, hypodense respect to the surrounding tissue in the basal phase (1) with a light peripherical enhanced rim in the arterial phase (2) that increases in the portal venous phase (3). In late phase (4), the lesion shows a puntacte aspect because of the presence of intralesional hypervascular spots (arrow). The bile ducts are not dilatated.

**Figure 2:**
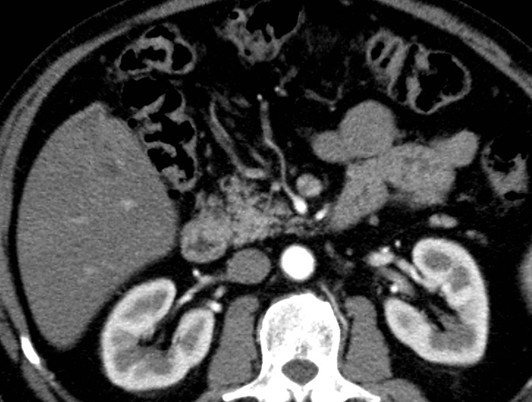
**Transverse CT scan**. It reveals a solid focal mass, about 3 cm in diameter, hypodense respect to the surrounding tissue in the basal phase (1) with a light peripherical enhanced rim in the arterial phase (2) that increases in the portal venous phase (3). In late phase (4), the lesion shows a puntacte aspect because of the presence of intralesional hypervascular spots (arrow). The bile ducts are not dilatated.

**Figure 3:**
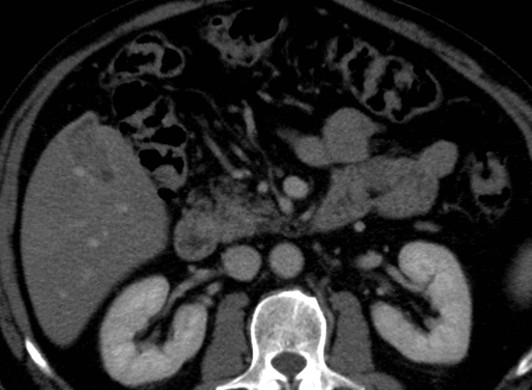
**Transverse CT scan**. It reveals a solid focal mass, about 3 cm in diameter, hypodense respect to the surrounding tissue in the basal phase (1) with a light peripherical enhanced rim in the arterial phase (2) that increases in the portal venous phase (3). In late phase (4), the lesion shows a puntacte aspect because of the presence of intralesional hypervascular spots (arrow). The bile ducts are not dilatated.

**Figure 4:**
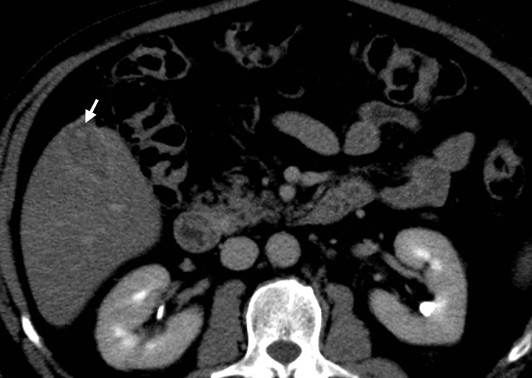
**Transverse CT scan**. It reveals a solid focal mass, about 3 cm in diameter, hypodense respect to the surrounding tissue in the basal phase (1) with a light peripherical enhanced rim in the arterial phase (2) that increases in the portal venous phase (3). In late phase (4), the lesion shows a puntacte aspect because of the presence of intralesional hypervascular spots (arrow). The bile ducts are not dilatated.

A 67-year-old Caucasian man underwent an ultrasound investigation because he suffered from right upper quadrant subcontinuous abdominal pain. The ultrasound examination and the successive Computed Tomography (CT)-examination, this last performed using an Aquilion 64 slice (Toshiba Medical System, Zoetmeer, Netherlands) revealed a solid, focal mass, about 3 cm in diameter, at the V hepatic segment in the presence of diffuse steatosis. On CT the mass presented hypodense respect to the surrounding tissue during the basal phase (Figure 1) and with a light peripherical enhanced rim in the arterial phase that increased in the portal venous phase. (Figure 2, 3) In late phase, the lesion showed a puntacte aspect because of the presence of intralesional hypervascular spots. (Figure 4) The bile ducts did not appear dilatated.

The patient was admitted to the medical department of our hospital for further investigation. On admission, physical examination and laboratory findings were normal (included negative hepatitis B and C – biomarkers), showing only a mild increase of alpha-feto-protein (73 IU/mL).

**Figure 5:**
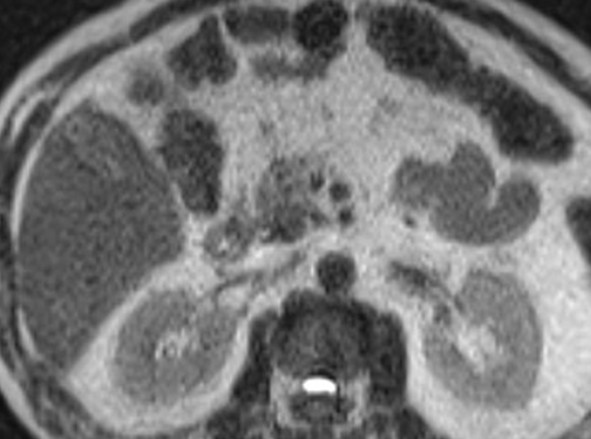
**Transverse RM scan**. It reveals a slightly dishomogeneous hyperintense mass in sequences with long TR (5) and the appearance of intralesional hypervascular spots (arrow) in late phase after administration of paramagnetic contrast agent (6).

**Figure 6:**
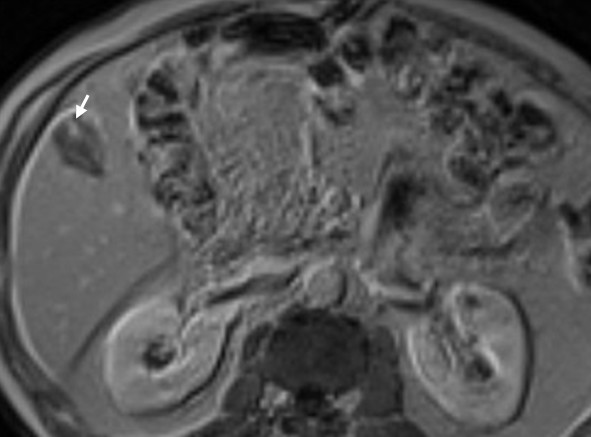
**Transverse RM scan**. It reveals a slightly dishomogeneous hyperintense mass in sequences with long TR (5) and the appearance of intralesional hypervascular spots (arrow) in late phase after administration of paramagnetic contrast agent (6).

At this time, the patient underwent a upper abdominal Magnetic Resonance (MR) investigation, performed with a Eclipse 1.5 T (Picker-Marconi, Philips, Eindhoven, Netherlands) which showed the mass hypointense in T1 W unenhanced images and slightly dishomogeneous hyperintense in sequences with long TR. (Figure 5) After administration of paramagnetic contrast agent, the lesion presented the same dynamic behaviour as on CT including the appearance of intralesional hypervascular spots in late phase. (Figure 6)

The lesion suggested different diagnostic hypotheses, from a peripherical cholangiocarcinoma to a primitive degenerated mass characterized by an unusual dynamic pattern or a dysplastic nodule with focal hepatocellular carcinoma and this last diagnostic hypothesis was confirmed by contrast enhanced ultrasound (CEUS).

Two different biopsies were performed using Biomol 21 G (H.S. Medical, USA), one revealed inconclusive and the other showed only the presence of a micronodular cirrhosis that was regarded associated to non-alcoholic steatohepatitis.

Finally, the patient was admitted to the surgical department of our hospital for the treatment.

The mass was therefore excised by segmentectomy of the V hepatic segment, and after that the patient had an uneventful postoperative course.

The liver lesion was 3 cm in the larger size; growing within a bile duct as a well demarcated large mass, no dilatation, stone or mucin were found in the large intrahepatic bile ducts around the lesion. Samples of the lesion were formalin fixed, paraffin embedded and stained with hematoxylin and eosin (H&E) and Alcian Blue and periodic acid Schiff stain (AB-PAS).

**Figure 7:**
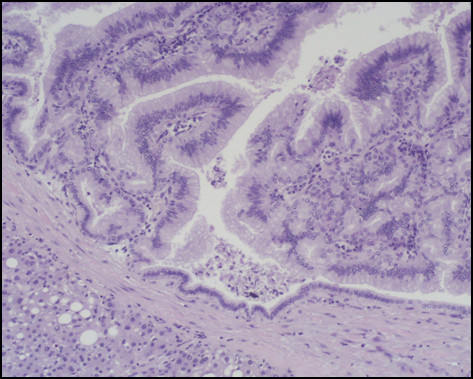
**Pathological sections specimens**. (7) Low magnification showing encapsulated papillary tumor with thin fibrovascular stalk, covered by tall mucinous moderately dysplastic epithelium (8). Higher magnification demonstrates area with oncocitic pattern (9). The immunohistochemical test showed a diffuse CK7+ (10) and apomucines MUC5AC (11) and MUC6 (12) positivity in more than 50% of neoplastic cells.

**Figure 8:**
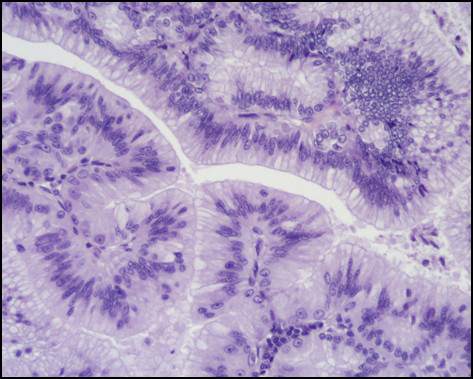
**Pathological sections specimens**. (7) Low magnification showing encapsulated papillary tumor with thin fibrovascular stalk, covered by tall mucinous moderately dysplastic epithelium (8). Higher magnification demonstrates area with oncocitic pattern (9). The immunohistochemical test showed a diffuse CK7+ (10) and apomucines MUC5AC (11) and MUC6 (12) positivity in more than 50% of neoplastic cells.

**Figure 9:**
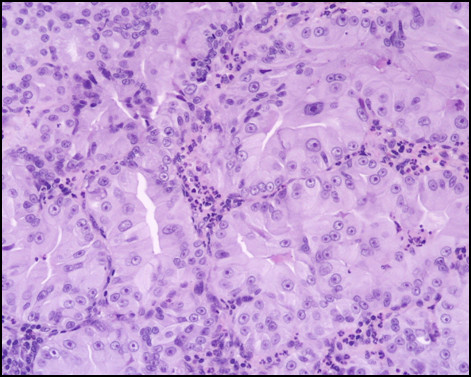
**Pathological sections specimens**. (7) Low magnification showing encapsulated papillary tumor with thin fibrovascular stalk, covered by tall mucinous moderately dysplastic epithelium (8). Higher magnification demonstrates area with oncocitic pattern (9). The immunohistochemical test showed a diffuse CK7+ (10) and apomucines MUC5AC (11) and MUC6 (12) positivity in more than 50% of neoplastic cells.

**Figure 10:**
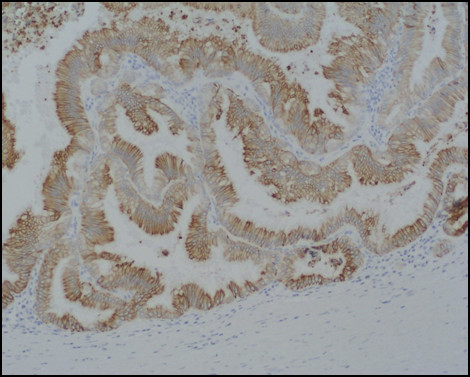
**Pathological sections specimens**. (7) Low magnification showing encapsulated papillary tumor with thin fibrovascular stalk, covered by tall mucinous moderately dysplastic epithelium (8). Higher magnification demonstrates area with oncocitic pattern (9). The immunohistochemical test showed a diffuse CK7+ (10) and apomucines MUC5AC (11) and MUC6 (12) positivity in more than 50% of neoplastic cells.

**Figure 11:**
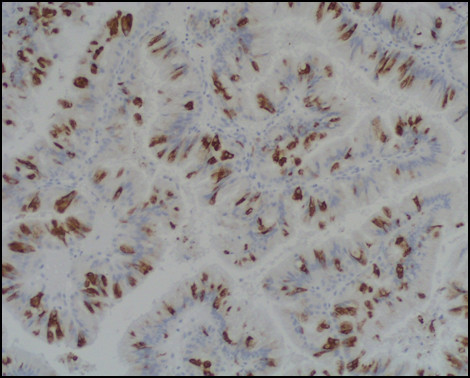
**Pathological sections specimens**. (7) Low magnification showing encapsulated papillary tumor with thin fibrovascular stalk, covered by tall mucinous moderately dysplastic epithelium (8). Higher magnification demonstrates area with oncocitic pattern (9). The immunohistochemical test showed a diffuse CK7+ (10) and apomucines MUC5AC (11) and MUC6 (12) positivity in more than 50% of neoplastic cells.

**Figure 12:**
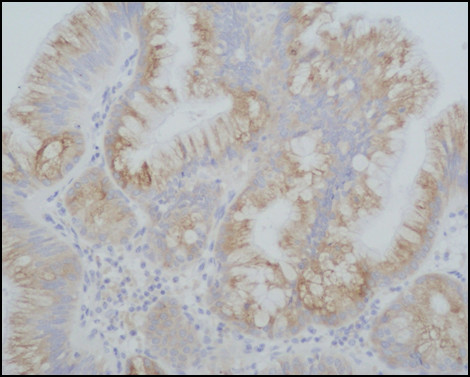
**Pathological sections specimens**. (7) Low magnification showing encapsulated papillary tumor with thin fibrovascular stalk, covered by tall mucinous moderately dysplastic epithelium (8). Higher magnification demonstrates area with oncocitic pattern (9). The immunohistochemical test showed a diffuse CK7+ (10) and apomucines MUC5AC (11) and MUC6 (12) positivity in more than 50% of neoplastic cells.

Immunostaining for MUC1, MUC2, MUC5AC, MUC6, CDX2, CK-7, and CK-20 were performed on sections specimens that were dewaxed and rehydrated using Bio-clear (Bio-Optica, Milan, Italy) and graded alcohols. Endogenous peroxidase was blocked dipping sections in 3% aqueous hydrogen peroxide for 10' and antigen retrieval was performed with 10' microwave treatment in 10 mM citrate buffer, pH 6.00. The immunostaining was performed with the avidin-biotin-peroxidase complex technique using diaminobenzidine as chromogen. The sections were incubated overnight at 4°C with monoclonal antibodies against CDX2-88 (Biogenex) raised against a full-length CDX2 recombinant protein at 1:100 dilution, CK7 (clone OV-TL 12/30; Dako Cytomation at 1:50 dilution) and CK20 (cloneKs.20.8; Dako Cytomation at 1:50 dilution). The apomucines were studied by using a mouse monoclonal antibodies against MUC-1 glycoprotein (MA695, Novacastra Lab., Newcastle, UK) at 1:100 dilution; MUC-2 glycoprotein (CCP58, Novacastra Lab., Newcastle, UK) at 1:200 dilution; MUC-5AC glycoprotein (CLH2, Novacastra Lab., Newcastle, UK) at 1:100 dilutionand MUC-6 glycoprotein (CLH5, Novacastra Lab., Newcastle, UK) at 1:100 dilution. The neoplasm was made of papillary proliferation with thin fibrovascular stalk, covered by mild to moderate dysplastic epithelium, with tall columnar cells, mainly of mucinous type. A limited area of the tumor, showing an oncocitic pattern, was made of cuboidal cell with acidophilic, granular cytoplasm and prominent nucleoli. No goblet cells were found. (Figure 7, 8, 9, 10, 11, 12)

The immunohistochemical test showed a diffuse CK7+/CK20- pattern of neoplastic cells. The apomucines MUC5AC and MUC6 were positive in more than 50% of neoplastic cells, indicating a gastric foveolar phenotype while CDX2 and MUC2 were negative, indicating that intestinal phenotype was completely absent; also MUC1 was negative. On the basis of these findings a diagnosis of borderline (gastric type) was made.

**Figure 13:**
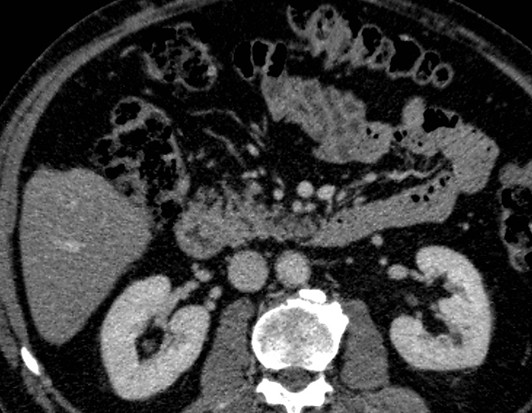
**CT follow up examination**. It shows the patient free of the disease.

The successive CT follow up examinations showed the patient free of the disease. (Figure 13)

## Discussion

Intraductal papillary mucinous tumor of the bile ducts is characterized by innumerable frondlike infoldings of proliferated columnar epithelial cells and slender fibrovascular stalks that are supported by connective tissue from the lamina propria [1,2]. At histopathologic analysis it may present as adenocarcinoma, adenoma, dyspalsia or as a mixed form [1].

Papillary cholangiocarcinoma rappresents 3–5% of the reported cholangiocarcinomas [6].

Papillary adenocarcinoma is a low-grade malignancy that grows slowly and tends to spread along the mucosal surface and invading the ductal wall in the late phase of the development [1,2,7].

Papillomatosis represents a known condition where tumors are multiple and extend along bile ducts [1,2].

Intraductal papillary tumor produces a variable amount of mucin that may be retained in the lesion. Some tumors produce a large amount of mucin that is discharged into the bile ducts and may intermittently and partially impede the flow of bile juice, causing obstructive jaundice, sometimes complicated by cholangitis or stone formation. Endoscopy may show mucin draining from the papilla of Vater. This condition is diagnostic of a mucin-producing tumor of the bile or pancreatic ducts. When the mucin flow obstructs the duodenal papilla, both proximal and distal to the tumor bile ducts become diffusely dilated. So dilatation can be caused by the presence of the lesion as by the mucin excretion. Dilatation may affect only the segmental or lobar bile ducts or the entire biliary tree depending on the location of the cause. Moreover, the dilatation in bile ducts containing tumor may be disproportionate with that in the surrounding biliary tree [1,2].

The clinical manifestations are consisting in upper abdominal pain, episodic biliary pain, fever and jaundice. These symptoms are related to the recurrent obstruction of the bile ducts [1].

The imaging techniques used for the study of papillary bile ducts neoplasms include ultrasonography (US), CT, endoscopic retrograde cholangiopancreatography (ERCP) and MR cholangiopancreatography that reveal bile ducts dilatation with or without visible intraductal papillary lesion [1,2,8].

Bile ducts that contain a mucinous intraductal papillary tumor may dilate diffusely or focally like an aneurysm and this presentation, such as the disproportionate lobar dilatation of the bile ducts where the tumor is localized than the ducts in the controlateral lobe, are considered a characteristic sign of the presence of an intraductal papillary mucinous tumor. So the involved bile ducts may dilate aneurysmlike, whereas others diffusely and proportionally [1,2].

Despite the study of Lim et al., where imaging techniques showed the presence of bile duct dilatation in all 15 patients, our case was not associated to bile duct dilatation, probably because the tumor was localized peripherically.

At US papillary tumor can appear as an echogenic mass. The echogenicity is similar to that of the liver and there is no acoustic shadow. In our case, the lesion appeared hypoechogenic in the presence of surrounding steatosis. On CT the neoplasm presents as intraluminal soft-tissue mass enhancing or nonenhancing depending on whether the tumor is connected to the wall or not. When it is fixed, it shows confined within the ducts and preserves the bile ducts wall integrity. Moreover, the tumor can present as asymmetric enhanced thickening of the bile duct wall [2,8]. Besides, the tumor may not be depicted when it is small and isoattenuating to the adjacent hepatic tissue or when the complex orientation of the dilated ducts obscures the mass [2].

Yoon et al, describing the CT findings in 15 patients affected by malignant papillary neoplasms of intrahepatic bile ducts, reported that most of the tumors were hypo- or isoattenuating on contrast-enhanced CT. Conversely, CT could reveal an intraductal hypoattenuating mass associated with markedly dilated intra- and extrahepatic bile ducts in the three patients with mucin-hypersecreting papillary cholangiocarcinoma [6].

In this case, the appearance of a mass showing hypovascular with the enhanced rim in later phases, suggesting the presence of a capsule, could invite to include the hepatocellular carcinoma with atypical features in the differential diagnosis. Early stages like a dysplastic nodule with focal degenerated areas was a inconsistent hypothesis because the focal hypervascular degenerated areas did not show in the arterial phase. The low attenuation of the lesion in the arterial and venous portal sequences and the elongated morphology, as in our case, could suggest the presence of a peripheral cholangiocarcinoma but, on the contrary, the late intralesional enhancement was poor [9]. Because a definitive diagnosis, based on imaging appearance of the lesion, was impossible in our case, we decided to perform a hystopathological investigation but the biopsies were inconclusive. So, the definitive diagnosis was made on surgical resected material.

The use of ERCP is recommended to detect the presence and the location of the suspected neoplasm and to reveal the presence and the quantity of mucin draining from duodenal papilla. Moreover, ERCP permits biopsy performation [1]. This last procedure could not be performed in our case because the tumor was peripherical.

On ERCP the lesion appears as filling defect representing papillary tumor or as fine irregularities of the bile duct wall with a velvety or serrated contour.

On US mucin is echo-free but may appear as a echogenic focus; on CT it is water-attenuating.

Instead, ERCP may show small or large, elongated or amorphous filling defects caused by mucin presence in the dilated ducts.

Various conditions present as filling defects in the dilated ducts and include air bubbles, stones, blood clots, and intraluminal tumors [2,10,11].

However, the papillary tumors of the bile ducts are characterized by the inconsistency and changeability of the imaging findings. Moreover, there can be differences between the radiologic and surgical presentation, and both these can differ from the pathologic findings but when intraductal masses are seen with localized dilatation of the intrahepatic bile ducts, papillary tumor of the bile ducts should be included in the differential diagnosis. Otherwise, the diagnosis may be made on the basis of histopathologic examination, as in our case [6,8].

## Abbreviations

IPNL: Intraductal papillary neoplasm of the liver; IPMT: Intraductal papillary mucinous tumor; CT: Computed Tomography; MR: Magnetic Resonance; CEUS: Contrast enhanced ultrasound; H&E: Hematoxylin and eosin; AB-PAS: Alcian Blue and periodic acid Schiff stain; US: Ultrasonography; ERCP: Endoscopic retrograde cholangiopancreatography;

## Consent

Written informed consent was obtained from the patient for publication of this case report and accompanying images. A copy of the written consent is available for review by the Editor-in-Chief of this journal.

## Competing interests

The authors declare that they have no competing interests.

## Authors' contributions

GC, EG analyzed and interpreted the patient data and the radiologic investigations. RD, GD performed the hepatic segmentectomy. FS performed the histological examination of the resected specimen and was a major contributor in writing the manuscript. EB was a major contributor in writing the manuscript. TC was a contributor in writing the manuscript. CF revised the manuscript. All authors read and approved the final manuscript.
